# Prevalence of External Injuries in Small Cetaceans in Aruban Waters, Southern Caribbean

**DOI:** 10.1371/journal.pone.0088988

**Published:** 2014-02-19

**Authors:** Jolanda A. Luksenburg

**Affiliations:** Department of Environmental Science and Policy, George Mason University, Fairfax, Virginia, United States of America; Aristotle University of Thessaloniki, Greece

## Abstract

Aruba, located close to the coasts of Colombia and Venezuela, is one of the most densely populated islands in the Caribbean and supports a wide range of marine-related socio-economic activities. However, little is known about the impacts of human activities on the marine environment. Injuries in marine mammals can be used to examine interactions with human activities and identify potential threats to the survival of populations. The prevalence of external injuries and tooth rake marks were examined in Atlantic spotted dolphin (*Stenella frontalis*) (*n = *179), bottlenose dolphin (*Tursiops truncatus*) (*n = *76) and false killer whale (*Pseudorca crassidens*) (*n = *71) in Aruban waters using photo identification techniques. Eleven injury categories were defined and linked to either human-related activities or natural causes. All injury categories were observed. In total, 18.7% of all individuals had at least one injury. Almost half (41.7%) of the injuries could be attributed to human interactions, of which fishing gear was the most common cause (53.3%) followed by propeller hits (13.3%). Major disfigurements were observed in all three species and could be attributed to interactions with fishing gear. The results of this study indicate that fishing gear and propeller hits may pose threats to small and medium-sized cetaceans in Aruban waters. Thus, long-term monitoring of population trends is warranted. Shark-inflicted bite wounds were observed in Atlantic spotted dolphin and bottlenose dolphin. Bite wounds of cookie cutter sharks (*Isistius* sp.) were recorded in all three species, and include the first documented record of a cookie cutter shark bite in Atlantic spotted dolphin. This is one of the few studies which investigates the prevalence of injuries in cetaceans in the Caribbean. Further study is necessary to determine to which extent the injuries observed in Aruba affect the health and survival of local populations.

## Introduction

In most parts of the world, marine mammals are exposed to various threats caused by interaction with human activities, including fisheries, boat traffic, contaminants and pathogens [1]–[4]. These human activities are increasing worldwide, especially in coastal areas [5], [6]. As a consequence, the conservation status of many species of marine mammals is of great concern [7]. Marine mammals are also subjected to natural threats, including predators such as killer whale *Orcinus orca*, false killer whale *Pseudorca crassidens* and a variety of shark species [8], [9]. Unfortunately, only few marine mammal populations and species have been studied sufficiently in terms of threat levels, which hinders assessment of their conservation status [10]–[12].

External injuries of marine mammals have provided useful information for a wide range of studies in ecology and conservation biology [13], [14]. Fishing gear, propellers and vessel collisions often leave distinctive wounds or scars which can be identified from photographs [11], [15], [16]. Consequently, the presence of such wounds and scars helps to establish to which human activities marine mammals are exposed. External injuries may also provide information about the interactions of marine mammals with members of their own species and other species. For instance, inter- and intraspecific aggressive behavior can be studied using the prevalence and location of tooth rakes [17], [18]–[22]. Interactions between sharks and dolphins may be inferred from the presence of shark-inflicted wounds [8], [23]–[25]. Because individual dolphins and whales often can be identified from their external markings [13], [26], it is possible to quantify the prevalence of injuries within a local population.

The prevalence of injuries has been reported for a small number of species in a small number of areas [14]–[16], [27]–[29]. However, most of these studies were limited to human-caused injuries and thus did not include injuries caused by inter- and intraspecific interactions. In addition, the majority of studies focused on the bottlenose dolphin *Tursiops truncatus* [16], [30], [31], which may not be representative of other species. Only a few studies have quantified injuries of multiple species inhabiting the same area, including Hong Kong, Mayotte (western Indian Ocean) and Peru [14], [27], [32]. To date, only one study has measured the prevalence of injuries of marine mammals in the Caribbean. Van Bressem et al. [27] reported a low prevalence of injuries (≤ 1.5%) in two samples of Atlantic spotted dolphins *Stenella frontalis* along the central coast of Venezuela.

The marine mammal fauna of the Caribbean Sea includes at least 29 species of cetaceans, most of which are poorly known or studied [33]–[35]. The Caribbean marine environment has been highly impacted by human activities [36], which may translate into a higher extinction risk of its marine mammals [12]. One species, the Caribbean monk seal *Monachus tropicalis*, has recently become extinct due to hunting and overfishing [37], [38]. Therefore, it is important to identify and quantify possible threats to marine mammal populations in the Caribbean.

Aruba is one of the most densely populated islands in the Caribbean and supports a range of marine-related socio-economic activities, including fishing, shipping, oil refinery and tourism. Recently, 16 species of Cetacea have been identified in the coastal waters of Aruba [39]–[42]. However, little is known about their threats and conservation status. Because Aruba is a small island with a short coastline, stranding incidents of cetaceans are rare. Therefore, by necessity research relies largely on photographic data collected at sea [42].

In this study, photographs were used to identify and categorize injuries, to measure their prevalence in three species observed at close distance to the Aruban coast, and to compare their prevalence with those previously reported for other populations and species worldwide.

## Methods

### Ethics Statement

All animals were observed and photographed from boats within their natural habitat in Aruban waters. No specific permissions were required for these locations/activities. The three species are not considered endangered (IUCN Red List of Threatened Species, Version 2013.1). All three species are protected by international conventions and agreements, including *International Convention for the Regulation of Whaling* (1946), *Convention on International Trade in Endangered Species of Flora and Fauna* (1973), *Convention for the protection and development of the marine environment of the Wider Caribbean Region* (1983), *Protocol on Specially Protected Areas and Wildlife* (1990), and *Convention on Biological Diversity* (1992). However, the animals were not killed, injured, captured, touched, sampled or harassed during the surveys. The study therefore complies with all relevant legislation and did not require approval from the Institutional Animal Care and Use Committee of George Mason University.

### Study Area

**Figure 1:**
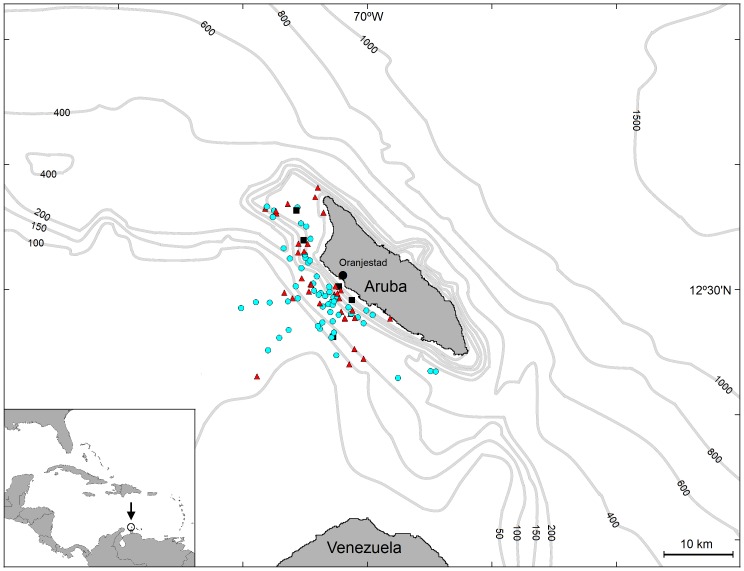
Study area and sighting locations. Sighting locations of Atlantic spotted dolphins (blue circles), bottlenose dolphins (red triangles) and false killer whales (black squares). Depth contours are in meters.

Aruba (12°30’N 69°58’W) is a small island (193 km^2^) located in the southern part of the Caribbean Sea at approximately 27 km North off the Paraguaná Peninsula of Venezuela (Fig. 1). The waters between Aruba and Venezuela are no more than 200 meters deep, whereas on the northeast side deep waters (>1000 m) are reached within 14 km of shore. Sea water temperature varies between 25°C in February and 28°C in September [43].

In Aruban waters, fisheries are artisanal. Fishing is typically conducted from small motorized boats (≤ 10 meter). Methods consist principally of handlining and/or trolling using up to seven lines with a maximum of four hooks per line. Other methods, used on a smaller scale, include the use of long seine nets and gillnets, traps and spear fishing (pers. obs.) [44], [45]. Aruban waters are used intensively for anchoring of large containerships, transport (containerships and cruise ships), fishing and recreation. An oil refinery (Valero) is present at the coast near the east point of the island.

### Data Collection

Cetacean surveys were conducted from 9 April 2010 to 22 November 2011 during the daytime (between 0600h and 1900h) in Beaufort 5 or less. In this study, only sightings made in Beaufort 4 or less were used. Observations were made from several sport fishing boats on a near-daily basis. Boat trips did not follow any predetermined route. Trips typically lasted 4 hours at an average speed of 12 km/h. All surveys were within 31 km from shore. A more detailed description of the methodology is given elsewhere [42]. When cetaceans were sighted, an attempt was made to photograph as many individuals as possible. Photographs were taken using a digital camera (Nikon D200) and a 70–300 mm zoom lens (Nikon 1:4.5–5.6 AF-S).

The presence of injuries was assessed in three species, Atlantic spotted dolphin, bottlenose dolphin, and false killer whale. Atlantic spotted dolphins and bottlenose dolphins were observed hunting, travelling, resting, milling and socializing in shallow waters (<250 m deep) close to the island’s north-west and south-west coasts (Fig. 1) [42]. Both species were encountered year-round in the coastal waters of Aruba [42]. False killer whales were observed hunting and travelling along the north-west and south-west coasts in shallow waters (<105 m deep) during April, July and December (Fig. 1). A more detailed description of the cetaceans in Aruban waters is given elsewhere [42].

Individuals were identified using marks on the dorsal fin and body and the shape of the dorsal fin [13]. The program Darwin (Eckerd College, St. Petersburg, FL) was used to help match photographs of individuals. Digital images of identifiable individuals were visually screened for tooth rakes and externally visible injuries. All nicks on the leading edge of the dorsal fin were regarded as injuries because of the sturdy structure of the leading edge in dolphins, and because nicks on the leading edge are believed to be probably the result of human activities such as fisheries [46]. The trailing edge of the dorsal fin may have many small nicks as a result of daily life. Therefore, nicks on the trailing edge were only considered to be injuries if these were deeper than 5%. The relative depth of the nick was determined by dividing the depth of the nick (as measured on a photograph) by the total length of the base of the dorsal fin. The total length of the dorsal fin base was measured between the anterior and posterior insertions of the dorsal fin.

### Injuries

**Figure 2:**
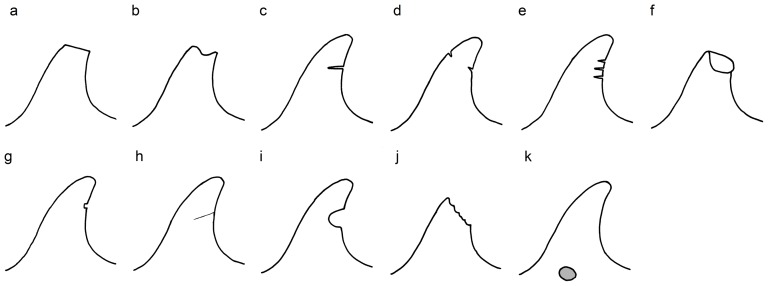
Injury categories. Illustrated are: (a) linear severed dorsal fin, (b) non-linear severed dorsal fin, (c) straight, deep cut, (d) opposing cuts, (e) parallel cuts, (f) partial collapse, (g) obtuse, short, cut-like indentation, (h) indentation, (i) round cut, (j) jagged, shark-inflicted bite wound, (k) cookie cutter shark bite wound.

Wounds and scars on the dorsal fin and body were classified into 11 categories, and were distinguished as follows:

- *Linear severed dorsal fin* (Fig. 2a): cleanly severed part of the dorsal fin. This type is most likely the result of interaction with human activities. Causes attributed to amputations with a clean linear surface include a knife cut [46], a propeller hit [11], [31], [47], and interactions with fishing gear, especially if the amputation is accompanied by scar tissue at the side(s) [14], [15], [48].
- *Non-linear severed dorsal fin* (Fig. 2b): non-cleanly severed part of the dorsal fin with irregular borders. This type is probably the result of inter- or intraspecific interaction [11], [14]. A jagged contour of the severed part is most likely the result of a shark attack [25].
- *Straight, deep cut* (Fig. 2c): wound characterized by a v-shaped cut which is more deep than high. This type of injury is likely caused by either fishing gear (lines/nets) cutting into the tissue [16], [46] or a propeller. Although a propeller injury is usually identified as a series of parallel, evenly spaced cuts [46], [49], there are examples of a single (diagonal) deep cut that were caused by a propeller [11], [16], [47]. Propeller cuts may be straight, curved or semi-curved incisions. The presence of a vertical incisive scar on the top of a dorsal fin is probably the result of a propeller hit [50].
- *Opposing cuts* (Fig. 2d): cuts or cut-like indentations on opposing sides of the dorsal fin (leading and trailing edge), flipper, tail or fluke. This type of injury is likely a result of human activities. For instance, opposing cuts may result from a fishing line which has been wrapped around the dorsal fin, leaving (deep) cut-like scars [16], [46], [48].
- *Parallel cuts* (Fig. 2e): multiple (straight, curved or semi-curved) incisions, cuts or slashes that are typically parallel and evenly spaced and of variable length. These wounds are likely the result of a turning propeller hit and are typically found on the dorsal surface of the body. The space between the cuts and length of the cut are indicators of the size of the propeller and its rotation speed [11], [46], [47], [49], [51]–[53].
- *Collapse* (Fig. 2f): dorsal fin that is completely or partially bent over. This may result from poor health or stress, or from entanglement with fishing gear [15], [20], [54]. If injuries are also present on the leading edge, fishing gear is believed to be a likely cause [15].
- *Obtuse, short, cut-like indentation* (Fig. 2g): wound characterized by a blunt cut-like indentation. This type is probably the result of interaction with either fishing gear [14], [46] or a propeller [16], [51], [55].
- *Indentation* (Fig. 2h): indentation or laceration in the epidermis, especially around the head, dorsal fin, flippers and fluke. This type likely results from entanglement in fishing gear (lines and/or nets) [16], [46], [48], [56].
- *Round cut* (Fig. 2i): half round or oval shaped cut, probably resulting from inter- or intraspecific interactions.
- *Shark-inflicted bite wound* (Fig. 2j): wounds or scars on the body that are either crescent-shaped (with or without puncture marks from teeth) or have deep and widely spaced tooth rakes [23]–[25]. On the dorsal fin, a jagged contour of the severed part is most likely the result of a shark attack [25].
- *Cookie cutter wound* (Fig. 2k): wound characterized by a small, circular, oval, elliptic or conical-shaped patch caused by a bite from a cookie cutter shark (*Isistius* sp.). When the wound is fresh the wound may resemble a crater of removed skin and blubber [8], [9], [57].

Wounds that could not be assigned to any of these categories were classified as *Unidentified*.

### Tooth Rakes (inter- and intraspecific)

Tooth rakes are parallel linear skin wounds or scars caused by the teeth of Odontoceti [21], [22]. Tooth rakes were classified as either new or old. New tooth rakes are characterized by broken skin, whereas old tooth rakes have either faint lines, black lines or white lines (scar tissue) [21]. No distinction was made based on coloration. The degree of coverage of tooth rakes was classified as (1) *major*: 50% or more of the dorsal side of the body (including head, anterior, dorsal fin, mid-flank, peduncle) is covered with tooth rake marks; (2) *medium*: less than 50% of the dorsal side of the body is covered, but the tooth rakes are still extensive and clearly visible; or (3) *minor*: only a few tooth rake marks are visible.

## Results

### Field Effort

Data were collected during 415 boat based surveys, covering 19,721 km of track line in the study area. Search effort covered all months of the year. During 88 boat based surveys, at least one of the three species was recorded. Atlantic spotted dolphins were photographed on 52 occasions, resulting in 179 identified individuals. In total, 40 individuals (23.3%) were resighted (seen two to six times). Bottlenose dolphins were photographed on 26 occasions and photographs from three additional sightings were obtained from third parties. In total, 76 identified individuals were included in the analyses. Twenty-six individuals (34.2%) were resighted (seen two to four times). False killer whales were photographed on 5 occasions, resulting in 71 identified individuals of which 21 individuals (29.6%) were resighted (seen two and three times). All three species were encountered close to shore (range 0.3–18.6 km) in water depths ranging from 13–550 m.

### Nicks in the Dorsal Fin

The majority of all individuals of the three species had nicks of variable size in the trailing and leading edges of their dorsal fin (Table 1). There was no significant difference among the three species in the prevalence of the total number of nicks (χ^2^ *= *1.08, df* = *2, ns) and in the prevalence of nicks in the leading edge (Fisher’s exact test; ns).

**Table 1:** Depth of dorsal fin nicks in Atlantic spotted dolphins, bottlenose dolphins and false killer whales in Aruban waters in 2010 en 2011.

Depth of nick^a^	*S. frontalis* (*n = *119)			*T. truncatus* (*n = *55)			*P. crassidens* (*n = *54)		
	Leading edge	Trailing edge	Both	Leading edge	Trailing edge	Both	Leading edge	Trailing edge	Both
≤ 5%	6	113	113	3	53	53	3	47	47
5–10%	2	5	5	-	2	2	-	7	-
≥ 10%	-	1	-	-	-	-	-	-	-

Only individuals with one or more nick(s) in their dorsal fin are presented.

a expressed as % of base of fin.

### Injuries

Injuries (*n = *72) were observed in 61 of 326 individuals (18.7%) of the three species combined (Table 2). The prevalence of injuries did not differ among the three species (χ^2^ *= *2.6, df* = *2, ns) (Table 2). All observed injuries, except two fresh wounds from cookie cutter sharks, were old, healed wounds (scars). Most injuries were located on the dorsal fin (62.5%), followed by the peduncle (23.6%), anterior body trunk (5.6%), fluke and flippers (5.6%), mid-flank (1.4%), and head (1.4%). In ten individuals, involving all three species, the dorsal fin was partially (*n = *9) or completely (*n = *1) amputated. There were as many linear as non-linear severed dorsal fins (Table 3).

**Table 2:** Prevalence of injuries in Atlantic spotted dolphins, bottlenose dolphins and false killer whales in Aruban waters in 2010 en 2011.

	*S. frontalis*	*T. truncatus*	*P. crassidens*
Injury type	(*n = *179)	(*n = *76)	(*n = *71)
**No injury**	149	63	53
**≥ 1 injury**	30 (37)	13 (15)	18 (20)
Dorsal fin injury only	17 (18)	10 (12)	12 (13)
Body injury only	11 (15)	3 (3)	5 (5)
Dorsal fin and body injuries	2 (4)	-	1 (2)
**With injuries probably human-related**	17 (21)^a^	3 (3)	6 (6)
Fisheries	8 (12)	2 (2)	2 (2)
Propeller	2 (2)	-	2 (2)
Fisheries or propeller	7 (7)	1 (1)	2 (2)
**With injuries probably natural**	9 (10)	7 (9)	7 (8)^b^
Bite mark, unidentified	7 (8)	5 (7)	5 (6)
Bite mark, shark	1 (1)	1 (1)	-
Bite mark, cookie cutter shark	1 (1)	1 (1)	2 (2)
**With unidentified injuries**	6 (6)	3 (3)	6 (6)

Numbers indicate individuals; numbers in parentheses indicate number of injuries.

a Two individuals had an injury caused by human activity and one with unknown cause.

b One individual had an injury caused by inter- or intraspecific interaction and one with unknown cause.

**Table 3:** Prevalence of injury types in Atlantic spotted dolphins, bottlenose dolphins and false killer whales in Aruban waters in 2010 en 2011.

	*S. frontalis* a	*T. truncatus* a	*P. crassidens* a
	*n = *30 (37)	*n = *13 (15)	*n = *18 (20)
Linear severed dorsal fin	2 (2)	2 (2)	1 (1)
Non-linear severed dorsal fin	3 (3)	2 (2)	-
Straight deep cut	5 (5)	1 (1)	3 (3)
Opposing cuts	2 (2)	-	-
Parallel cut	1 (1)	-	1 (1)
Collapse	-	-	1 (1)
Obtuse, short, cut-like indentation	5 (5)	-	-
Indentation	6 (6)	1(1)	-
Round cut	6 (6)	2 (4)	5 (6)
Shark-inflicted bite wound body	-	1 (1)	-
Cookie cutter shark wound	1 (1)	1 (1)	2 (2)
Unidentified	6 (6)	3 (3)	6 (6)

Numbers indicate individuals; numbers in parentheses indicate number of injuries.

a Individuals can have more than one type of injury.

In total, 30 injuries (41.7%) involving 26 individuals (42.6%) were likely the result of human activities. Of the animals with an injury, there was no significant difference among the three species in the proportion of human-related injuries (Fisher’s exact test, ns). The majority of human-related injuries was likely related to entanglement in fishing gear (53.3%) and to a lesser extent to propeller hits (13.3%) (Table 2).

In total, 27 injuries (37.5%) involving 23 individuals (37.7%) were likely the result of inter- or intraspecific interactions. The cause of most injuries, including round or oval-shaped cuts and non-linear severed dorsal fins, could not be attributed to any particular species. In total, there were six injuries indicating a shark attack, including cookie cutter bite wounds (*n = *4) and shark bite wounds (*n = *2) (Table 2).

The injuries of 15 individuals were classified as ‘unidentified’. Four of these were possibly inflicted by sharks (Table 2).

***Stenella frontalis.*** Among 179 Atlantic spotted dolphins, 30 (16.8%) individuals showed a total of 37 injuries (56.7% dorsal fin, 36.7% body, 6.7% both dorsal fin and body). Seven individuals had two injuries and 23 individuals had one injury (Table 2, 3).

**Figure 3:**
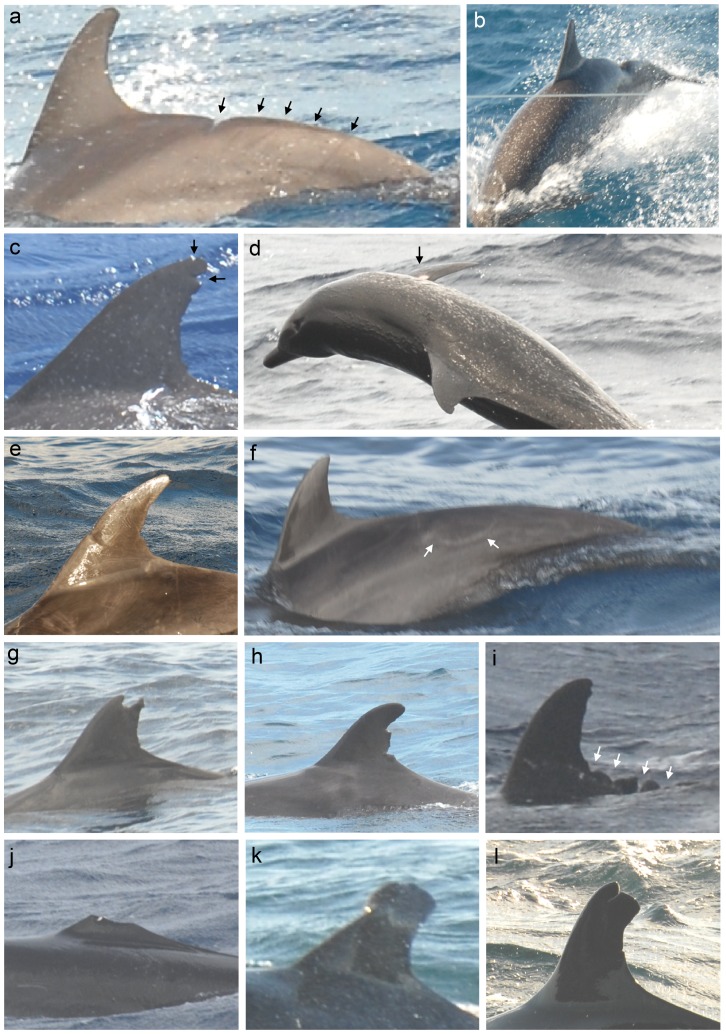
Injuries in Aruban cetaceans. Injuries include: (a) cut-like indentation and indentation injuries on the peduncle of an Atlantic spotted dolphin, (b) curvilinear incised scar, from left to right across its peduncle and extending to its left flank in an Atlantic spotted dolphin, (c) opposing cuts in the dorsal fin of an Atlantic spotted dolphin, (d) cookie cutter shark wound on the right flipper of an Atlantic spotted dolphin, (e) indentation marks on the dorsal fin of a bottlenose dolphin, (f) Scar of a healed shark-inflicted wound on the anterior peduncle of a bottlenose dolphin, (g) non-linear severed dorsal fin of a bottlenose dolphin, (h) oval shaped cuts in the trailing edge of a bottlenose dolphin, (i) parallel cuts behind the dorsal fin of a false killer whale, (j) linear severed dorsal fin with scar material (white) on either side of the missing fin of a false killer whale), (k) partial collapse of the dorsal fin of a false killer whale, (l) straight, deep vertical incisive scar on top of the dorsal fin of a false killer whale.

Of the 30 individuals with injuries, 17 individuals (56.7%) had at least one injury of probable anthropogenic origin, representing six injury types (Table 3, Fig. 3a-c). Eight individuals had injuries that were most likely caused by fishing gear, including two with opposing cuts in the dorsal fin, two with indentation marks, and four with a combination of injury types on their body (straight, deep cut or cut-like indentation in combination with an indentation). Two individuals had injuries that were most likely the result of a propeller hit, including parallel v-shaped cuts in the fluke (one individual) and a large, deep curvilinear incised scar on the body (one individual, see below). Seven individuals had injuries that were most likely caused by either fishing gear or a propeller hit, including two individuals with linear severed dorsal fins, two with v-shape cuts and three with cut-like indentations.

Injuries of probable natural origin were recorded in nine individuals (Table 2, 3). These included three different injury types, including one individual with a wound caused by a cookie cutter shark (Fig. 3d).

Six individuals had unidentified injuries which could not be attributed to either an anthropogenic or natural origin (Table 3).

Three individuals had major disfigurements. One individual had a non-linear severed dorsal fin with a jagged contour, and had lost approximately 40% of its dorsal fin, probably due to a shark attack. The second individual had a large, deep cut in its trailing edge with a squarish contour. The cause of this injury is unknown. It is possible that this injury represents a bite wound caused through an inter- or intraspecific interaction. The third individual had a large, deep curvilinear incised scar, from left to right across its peduncle and extending to its left flank towards its anterior (Fig. 3b). This injury was most likely caused by a propeller, probably a large and slowly rotating one.

***Tursiops truncatus.*** Among 76 identified bottlenose dolphins, 13 individuals (17.1%) showed 15 injuries (76.9% dorsal fin, 23.1% body) (Table 2, 3).

Of the 13 individuals with injuries, three individuals had one injury of probable anthropogenic origin (Table 2, 3). One individual showed indentation markings in the leading edge of the dorsal fin that probably were caused by fishing gear (Fig. 3e). In two individuals, the dorsal fin showed linear cuts, of which one was probably caused by fishing gear (see below) and the other could have been caused by either fishing gear or a propeller hit.

Injuries of probable natural origin were recorded in seven individuals (Fig. 3f-h, Table 2, 3). These included five different injury types, including one individual with two fresh cuts in the leading edge of the dorsal fin that were the result of teeth (toothrake marks).

Three individuals had single unidentied injuries which could not be attributed to either an anthropogenic or natural origin (Table 2, 3). One of these individuals appeared to have a crescent-shaped scar on its body (peduncle) which was possibly inflicted by a shark. However, the photograph was of insufficient quality to confirm this.

One bottlenose dolphin showed a major disfigurement: a linear severed dorsal fin with almost 40% of the fin missing. The presence of scar tissue along the severance indicated that the injury was not a congenital defect and likely resulted from fishing gear.

***Pseudorca crassidens.*** Among the 71 false killer whales, 18 individuals (25.4%) showed a total of 20 injuries (66.7% dorsal fin, 27.8% body, 5.6% both dorsal fin and body) (Table 2, 3).

Of the 18 individuals with an injury, six (33.3%) showed injuries that were probably caused by human activities (Fig. 3i-l, Table 2, 3). Two of these injuries were most likely caused by fishing gear (see below) and two injuries were most likely the result of a propeller hit. One of these had parallel, evenly spaced cuts at the base of its trailing edge extending to its anterior peduncle (Fig. 3i), and the other had a deep, narrow vertical incisive scar cut splitting the top of the dorsal fin (Fig. 3l). Two injuries of straight deep cuts were likely caused by either fishing gear or propeller hit.

Seven individuals (38.9%) had one injury of probable natural origin. These injuries include dorsal fins with very round or oval shaped scars (5 individuals) and bite wounds typical of that of a cookie cutter shark (2 individuals).

Six individuals (22.2%) had an unidentified injury of which the cause is unknown. These include three individuals that appeared to have crescent-shaped scars on their body which were possibly inflicted by sharks. However, the photographs were of insufficient quality to confirm this.

Two individuals (11.1%) had major disfigurements. One individual had a completely severed dorsal fin with a linear cut and scar marks on both sides of the cutting edge (Fig. 3j). Another showed a collapsed dorsal fin which was bent halfway of the fin (Fig. 3k). The sharp bent on the leading edge of the dorsal fin indicated that the collapse was caused by a fishing line.

### Tooth rakes

Tooth rake marks were recorded in individuals of all three species (Table 4). The number of individuals with tooth rake marks was significantly higher in bottlenose dolphins than in Atlantic spotted dolphins (Fisher’s exact test; *P<*0.001) and false killer whales (Fisher’s exact test; *P<*0.001) and significantly lower in Atlantic spotted dolphins than in false killer whales (Fisher’s exact test; *P<*0.01).

**Table 4:** Prevalence of tooth rake marks in Atlantic spotted dolphins, bottlenose dolphins and false killer whales in Aruban waters in 2010 en 2011.

	*S. frontalis*	*T. truncatus*	*P. crassidens*
	(*n = *179)	(*n = *76)	(*n = *71)
Individuals with tooth rake marks	52 (29.1%)	71 (93.4%)	35 (49.3%)
Minor coverage of tooth rake marks	49	38	35
Medium coverage of tooth rake marks	3	20	0
Major coverage of tooth rake marks	0	13	0
Old tooth rake marks	52	69	28
New tooth rake marks	1	9	7

The severity of the rake marks differed among the three species. In both Atlantic spotted dolphins and false killer whales, rake marks had a small area of coverage, whilst in bottlenose dolphins there was a high variability in the area covered by rake marks (Table 4).

**Figure 4:**
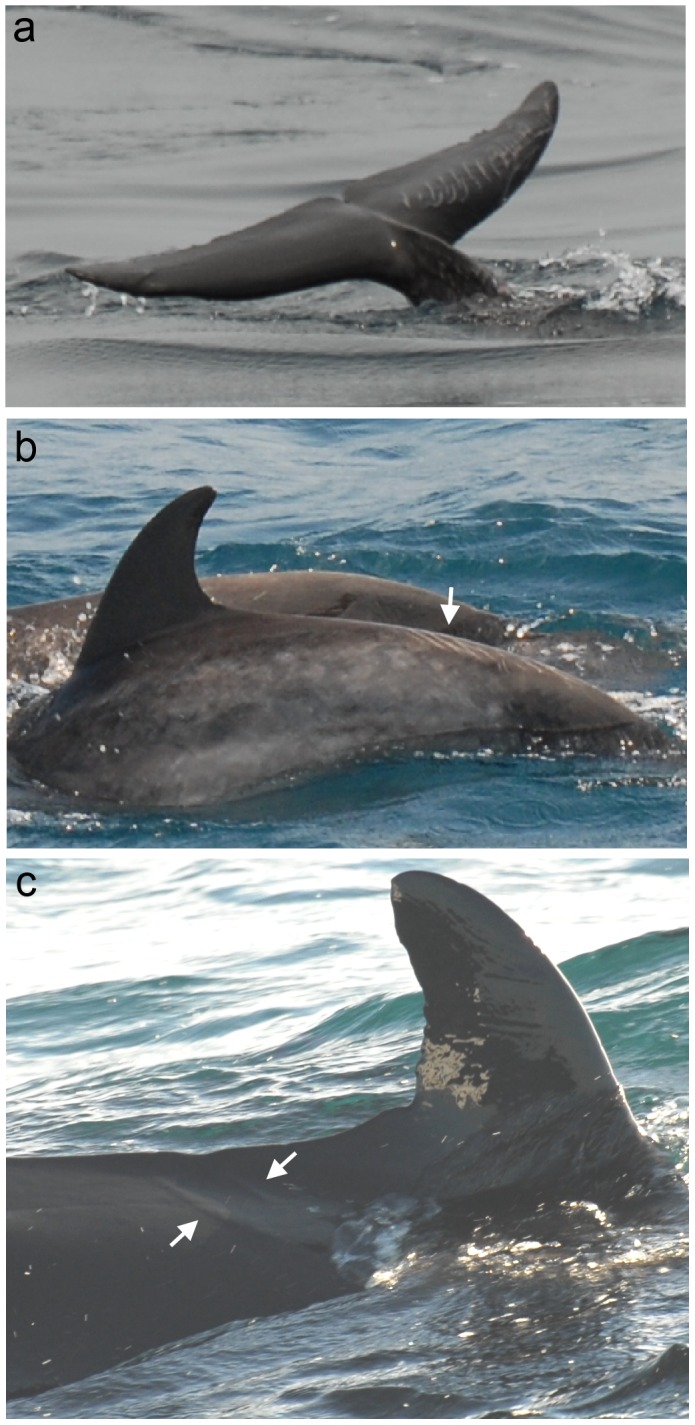
Tooth rake marks likely resulting from interspecific interactions. Illustrated are: (a) tooth rakes on the fluke of an Atlantic spotted dolphin, (b) tooth rakes on the tail stock of a bottlenose dolphin, (c) tooth rakes behind the dorsal fin of a false killer whale.

In each species, one individual was observed with tooth rake marks likely resulting from interactions with another species (Fig. 4). In each of these cases, the parallel rake marks were wider apart than the majority of observed rake marks.

## Discussion

### Comparison among the three species

This study documents injuries in three species of dolphins in Aruba. The proportion of individuals showing injuries was similar among the three species (16.8–25.4%). All three species showed injuries caused by both natural and anthropogenic sources and the relative contribution of these sources did not differ among the three species. The lack of differences between bottlenose dolphin and Atlantic spotted dolphin in both the proportion and cause of injuries is not surprising, given that both species co-exist and are found year-round in Aruban waters [42]. Thus, in Aruban waters these species are likely exposed to the same natural and human sources of injuries. False killer whales, on the other hand, were sighted only a few times during the study period, and thus most likely have a larger distribution range than the other two species, and are perhaps subjected to a larger set of potential sources of injuries. In addition, longevity of false killer whales is higher than that of bottlenose dolphins (57–65 years vs. 40–50 years [58]) and therefore false killer whales may be expected to accumulate more scars during their lifetime than the other two species. No detailed studies have been carried out which compare the prevalence and causes of injuries among different species. A full understanding of the vulnerability of different species to human threats requires long-term monitoring of population trends in species which co-exist in an area.

### Injuries caused by fishing gear

Most human-related injuries in the three species were likely due to interaction with fishing activities. Fishing gear was a likely cause of at least six types of injuries observed in Aruban waters. Although there are no direct observations of incidents between fisheries and cetaceans in Aruban waters, there are several indications that such incidents occur. During the study period, fishing was practiced year-round on a daily basis in the same areas where the three species were observed. All three species were observed at very close proximity to fishing boats and Atlantic spotted dolphins and bottlenose dolphins were observed interacting with the fishing lines. Furthermore, in August 2010 I observed and photographed the catch of a hammerhead shark (Sphyrnidae) in a gill net by a small fishing boat in an area where both Atlantic spotted dolphins and bottlenose dolphins were frequently observed [42]. Therefore, it seems likely that Atlantic spotted dolphins and bottlenose dolphins may also get entangled in such nets.

The proportion of probable fishery-related injuries recorded in the three species in Aruba (2.6–4.5%) was similar or higher than that documented for other species of dolphins in most previous studies: long-beaked common dolphin (*Delphinus capensis*) in Peru (0.6% [27]), Atlantic spotted dolphins in Venezuela (Central coast) (1.5% [27]), melon-headed whales (*Peponocephala electra*) in Mayotte, western Indian Ocean (1.6% [14]), Indo-Pacific humpback dolphins (*Sousa chinensis*) in Hong Kong (2.3% [59]), and short-finned pilot whales (*Globicephala macrorhynchus*) in Mayotte (4.4% [14]). However, the proportion of injuries in Aruba was lower than that reported for Guiana dolphin (*Sotalia guianensis*) in Brazil (5.0% [28]; 9.0% [29]), offshore common bottlenose dolphins in Peru (7.7% [27]) and Indo-Pacific bottlenose dolphins (*Tursiops aduncus*) around Mayotte (19.1% [14]).

This study documents a missing dorsal fin and a partially collapsed dorsal fin in false killer whales. Both injuries were most likely the result from interaction with fishing gear. In the individual with the missing dorsal fin, scar tissue was present at the cutting edge which suggests past contact with a sharp object, such as a fishing line. The individual with the collapsed dorsal fin showed a vertical cut-like injury at the leading edge of the dorsal fin at the location of the collapse, again indicating contact with a sharp object. In false killer whales, similar injuries have been reported only from Hawaii, which have been interpreted as a result of interactions with longline fisheries [15]. Although longlines are not used in Aruban waters, they are being used in nearby Venezuela and Colombia [60]–[62]. Given that false killer whales are not present year-round in Aruban waters, it seems likely that they have interacted with long-line fisheries elsewhere in the Southern Caribbean, perhaps in Venezuela or Colombia.

### Injuries caused by a propeller hit

Injuries caused by a propeller hit were observed in two Atlantic spotted dolphins and two false killer whales. Small propellers were the most probable cause of the injuries in both false killer whales, and one of the Atlantic spotted dolphins. The injuries of the other Atlantic spotted dolphin were consistent with a large, slow moving propeller. In the areas where the three species were observed, vessel traffic is intense, ranging from jet skis, speed boats to oil tankers. Boat traffic thus may pose a threat to cetaceans in Aruban waters. The prevalence of propeller hit injuries in Aruban waters was similar to that reported in previous studies in Florida and Hong Kong, two areas with heavy boat traffic. In Sarasota Bay, Florida, 3% of the bottlenose dolphins show injuries consistent with a propeller hit [16]. In Hong Kong, 2.8% of Indo-Pacific humpback dolphins show injuries caused by propeller hits [59]. Another study on the east coast of Florida found that 6% of the bottlenose dolphins showed evidence of a boat hit, but this included not only hits by a propeller but also collisions with other parts of the boat [30].

### Injuries caused by tooth rake marks

In Aruba, most bottlenose dolphins showed tooth rake marks (93.4%). The prevalence of tooth rake marks in bottlenose dolphins in Aruban waters was similar to that reported for bottlenose dolphins on the east side of Peron Peninsula in Australia (83% [21]). Tooth rake marks resulting from interaction with conspecifics are believed to be common among odontocetes [17]. However, this study provides the first estimates of the prevalence of tooth rake marks in Atlantic spotted dolphins (29.1%) and false killer whales (49.3%). The lower proportion of individuals with tooth marks in Atlantic spotted dolphins and false killer whales than in bottlenose dolphins suggests that these species show less aggressive or less frequent interactions among conspecifics than bottlenose dolphins.

In three individuals, the tooth rake marks appeared wider than what would be expected if these were caused by conspecifics (Fig. 4). The wide space between the rake marks on the Atlantic spotted dolphin (Fig. 4a) and the bottlenose dolphin (Fig. 4b) could indicate that the rake marks were caused by a larger species, perhaps false killer whale. The even wider rake marks on the false killer whale (Fig. 4c) were likely inflicted by a killer whale.

### Injuries caused by sharks

Crescent-shaped and jagged wounds typical of sharks bites were observed in one Atlantic spotted dolphin and one bottlenose dolphin. Three false killer whales had injuries that may have been caused by sharks but other causes could not be ruled out. All wounds were located on the dorsal part of the dolphin, which is consistent with other studies [24], [25]. It has been suggested that odontocetes turn their back to the shark when they are attacked or that odontocetes with attacks on their dorsal side are more likely to survive the attack [25]. There are at least ten shark species which are known to predate on cetaceans [25] and six of these occur in the Caribbean, including tiger shark (*Galeocerdo cuvier*), bull shark (*Carcharhinus leucas*), sixgill shark (*Hexanchus griseus*), dusky shark (*Carcharhinus obscurus*), oceanic white-tipped shark (*Carcharhinus longimanus*) and mako shark (*Isurus oxyrinchus*) [63], [64]. It is not known which of these species occur in Aruban waters. In any case, these records indicate that local dolphins may be subject to shark predation. More detailed studies of the impact of sharks on dolphins in the southern Caribbean may provide insights into the habitat use, group size and behavior of dolphins [8], [25].

Bite wounds from cookie cutter sharks (*Isistius* sp.) were recorded in all three species. Wounds from cookie cutter sharks have been documented in 49 species of cetaceans worldwide [57], including bottlenose dolphin and false killer whale. The bite wound of a cookie cutter shark in an Atlantic spotted dolphin (Fig. 3d) represents the first documented record for this species [57]. The presence of cookie cutter sharks in the Caribbean has been unclear. A fresh wound consistent with the bite of a largetooth cookie cutter shark (*Isistius plutodus*) has been recorded from a Cuvier’s beaked whale (*Ziphius cavirostris*) in Puerto Rico, and several old cookie cutter scars in cetaceans in Puerto Rico were hinted at but not specified [65]. Cookie cutter shark wounds have been recorded in the Lesser Antilles in Clymene dolphin (*Stenella clymene*) [66], [67] and rough-toothed dolphin (*Steno bredanensis*) [68] and in a dwarf sperm whale (*Kogia sima*) in Isla Marguerita, Venezuela [69]. Oval scars on a Gervais’ beaked whale (*Mesoplodon europaeus*) in Curaçao may have been from cookie cutter sharks [70]. These records make the presence of cookie cutter sharks likely in the southern Caribbean.

## Conclusion

This study is the first of its kind in the Caribbean, and underscores that photo-identification techniques may provide useful insights into anthropogenic threats in Caribbean populations of marine mammals. It represents one of the few quantitative studies of injuries in Atlantic spotted dolphins and false killer whales [11], [15], [27] worldwide and it adds to the growing literature on the injuries of bottlenose dolphins. This study may serve as a baseline for future surveillance of injuries in the Aruban populations of these species, for comparisons with offshore populations [71] and for comparison with other coastal areas in the Caribbean.

This study shows that the three species are exposed to and physically interact with boat traffic and fisheries, and thus may be at risk from human activities. Unfortunately, little information exists on the impact of injuries such as those observed in the present study on the long-term health, reproduction and survival of small cetaceans. Furthermore, although all the observed injuries were healed wounds on dolphins that survived, it is likely that more severe (e.g. fatal) injuries also occur but are overlooked. Thus, further study is necessary to document the proportion of fatal injuries and to determine to which extent the injuries observed in Aruba affect the health and survival of the local populations.
